# Prediction of Short-Term Mortality With Renal Replacement Therapy in Patients With Cardiac Surgery-Associated Acute Kidney Injury

**DOI:** 10.3389/fcvm.2021.738947

**Published:** 2021-10-21

**Authors:** Huiyong Han, Ziang Wen, Jianbo Wang, Peng Zhang, Qian Gong, Shenglin Ge, Jingsi Duan

**Affiliations:** Department of Cardiovascular Surgery, First Affiliated Hospital of Anhui Medical University, Hefei, China

**Keywords:** cardiac surgery, critical care score, mortality prediction, acute kidney injury, renal replacement therapy

## Abstract

**Objective:** We aimed to: (1) explore the risk factors that affect the prognosis of cardiac surgery-associated acute kidney injury (CS-AKI) in patients undergoing renal replacement therapy (RRT) and (2) investigate the predictive value of the Acute Physiology and Chronic Health Evaluation (APACHE) III score, Sequential Organ Failure Assessment (SOFA) score, and Vasoactive-Inotropic Score (VIS) for mortality risk in patients undergoing RRT.

**Methods:** Data from patients who underwent cardiac surgery from January 2015 through February 2021 were retrospectively reviewed to calculate the APACHE III score, SOFA score, and VIS on the first postoperative day and at the start of RRT. Various risk factors influencing the prognosis of the patients during treatment were evaluated; the area under the receiver operating characteristics curve (AUC_ROC_) was used to measure the predictive ability of the three scores. Independent risk factors influencing mortality were analyzed using multivariable binary logistic regression.

**Results:** A total of 90 patients were included in the study, using 90-day survival as the end point. Of those patients, 36 patients survived, and 54 patients died; the mortality rate reached 60%. At the start of RRT, the AUC_ROC_ of the APACHE III score was 0.866 (95% CI: 0.795–0.937), the VIS was 0.796 (95% CI: 0.700–0.892), and the SOFA score was 0.732 (95% CI: 0.623–0.842). The AUC_ROC_-value of the APACHE III score on the first postoperative day was 0.790 (95% CI: 0.694–0.885). After analyzing multiple factors, we obtained the final logistic regression model with five independent risk factors at the start of RRT: a high APACHE III score (OR: 1.228, 95% CI: 1.079–1.397), high VIS (OR: 1.147, 95% CI: 1.021–1.290), low mean arterial pressure (MAP) (OR: 1.170, 95% CI: 1.050–1.303), high lactate value (OR: 1.552, 95% CI: 1.032–2.333), and long time from AKI to initiation of RRT (OR: 1.014, 95% CI: 1.002–1.027).

**Conclusion:** In this study, we showed that at the start of RRT, the APACHE III score and the VIS can accurately predict the risk of death in patients undergoing continuous RRT for CS-AKI. The APACHE III score on the first postoperative day allows early prediction of patient mortality risk. Predictors influencing patient mortality at the initiation of RRT were high APACHE III score, high VIS, low MAP, high lactate value, and long time from AKI to the start of RRT.

## Introduction

In recent years, renal replacement therapy (RRT), as an important part of the treatment for critically ill patients, has become an established treatment modality for cardiac surgery-associated acute kidney injury (CS-AKI). Despite the increasing maturation of dialysis treatment techniques, cardiac surgery-associated AKI still carries a high mortality rate. The rate of AKI requiring concomitant RRT after cardiac surgery is <6% (1). For patients with AKI after a heart operation who require continuous dialysis, there is an eight-fold increase in mortality (2). Numerous studies show improved outcomes with an earlier initiation of continuous RRT (3, 4). Many studies have focused on the choice of early treatment timing and the progress of RRT (5–7). However, the choice of treatment timing is based on the “clinical judgment” of the physician. Cardiac surgery-associated acute kidney injury is mostly accompanied by damage to the liver, lungs, and other organs and is based on the severity of the disease as well as organ function. Hemodynamic changes seem to be more indicative of the condition, and as the timing of choice for RRT, the current Sequential Organ Failure Assessment (SOFA) score and vasoactive-inotropic score (VIS) have been used as early evaluation indicators of AKI after cardiac surgery. The Acute Physiology and Chronic Health Evaluation (APACHE) III score is currently the most commonly used score to assess the severity of illness in patients admitted to the intensive care unit (ICU). This study mainly focused on two aspects: (1) the risk factors of death in patients with CS-AKI as a basis for perioperative renal organ protection and the reduction of mortality in hospitalized patients, and (2) the value of the APACHE III score, VIS, and SOFA score in predicting the death risk of RRT in patients with CS-AKI.

## Materials and Methods

### Patient Population

This was a retrospective analysis. Data from a total of 90 patients who received RRT because of CS-AKI at the First Affiliated Hospital of Anhui Medical University from January 2015 through February 2021 were collected. The treatment outcomes were divided into survival and death groups by 90 days' survival after surgery. Cardiovascular surgery including coronary artery bypass grafting (CABG), off-pump CABG, heart valve surgery, aortic surgery, etc. The 90 days' survival rate was the primary endpoint, and secondary outcomes were multiple organ dysfunction syndrome (MODS) at discharge, blood urea nitrogen at discharge, creatinine at discharge, and duration of intensive care. Multiple organ dysfunction syndrome was defined by the occurrence of a total SOFA score >5, affecting two or more organs (8). The definition of CS-AKI: acute kidney injury occurring within 2 weeks after cardiac surgery (9). Considering that the prognosis of CS-AKI treated with RRT is a very complex pathophysiological outcome with many confounding factors. In order to exclude the interference of renal injury caused by preoperative non-surgery related factors on the mortality outcome as much as possible, we included patients developed acute kidney injury 2 weeks after surgery and were on RRT. Exclusion criteria: previous chronic kidney disease (CKD) or a postoperative survival time of <24 h. Chronic kidney disease was diagnosed when any of the following conditions exceeded 3 months: (1) Proteinuria (proteinuria excretion rate ≥30 mg/24 h); (2) Urine microprotein creatinine ratio ≥3 mg/mmol; (3) Abnormal urine sediment microscopy; (4) Renal tubular dysfunction leading to abnormal electrolytes or other abnormalities; (5) Abnormal renal histology; (6) Abnormal renal structure on imaging; (7) History of renal transplantation; (8) Decreased glomerular filtration rate <60 ml/(min·1.73m^2^) (10, 11). The timing of RRT was determined by consensus between the cardiac surgeon and the hemodialysis department. The primary indication was a postoperative creatinine value and (or) postoperative urine output that meets the 2012 Kidney Disease: Improving Global Outcomes (KDIGO) criteria for AKI (12) requiring measures such as volume expansion, inotropy, pressor use, and furosemide use. Renal replacement therapy patterns mainly consisted of continuous renal replacement therapy (CRRT) and intermittent renal replacement therapy (IRRT). Vascular access was established by indwelling single needle dual lumen tubing from the femoral vein using either continuous venovenous hemodiafiltration or continuous venovenous hemofiltration. Dialysate vs. replacement fluid is used after predialysis with a fixed formula and the mode of anticoagulation is decided based on the patient's bleeding risk, which was chosen from three modalities: no anticoagulation, systemic anticoagulation (unfractionated heparin or low molecular weight heparin) or regional anticoagulation (sodium citrate). Criteria for non-anticoagulant regimens: platelet count <50,000/μl, spontaneous or heparin related bleeding, basal activated partial thromboplastin time (APTT) <45 s, or surgery within the previous 48 h (13). According to AKI improving global outcomes (KDIGO) clinical practice guidelines, the application of regional anticoagulation, regardless of coagulation status, is extended to all patients undergoing CRRT with no contraindications for citrate (12). In addition, filter coagulation over a 24-h run time serves as a regional anticoagulation criterion. Patients with a greater bleeding tendency were anticoagulated without heparin, and coagulation was measured regularly after the initiation of treatment. The APTT remained stable until the target was 1.0–1.5 times the normal value.

### Data Collected

Basic patient information was collected from the medical record system of the First Affiliated Hospital of Anhui Medical University. Preoperative data comprised age, sex, body mass index (BMI), presence of hypertension, diagnosis of diabetes mellitus, history of cardiac surgery, and preoperative studies and routine blood work such as blood pressure, left ventricular ejection fraction, and hepatic and kidney function indicators. Intraoperative data included type of surgery, operative time, cardiopulmonary bypass time, cross clamp time, intraoperative urine volume, and blood transfusion information. Early morning indicators on the first day after surgery included: hemoglobin, white blood cells, platelets, albumin, total bilirubin, creatinine, urea nitrogen, glomerular filtration rate, C-reactive protein; The most recent indicators before RRT included: hemoglobin, white blood cells, platelets, total bilirubin, creatinine, urea nitrogen, glomerular filtration rate, C-reactive protein, mean arterial pressure (MAP); Other postoperative data collection included: the lowest MAP in the first 24 h after surgery, peak lactate before RRT, duration of mechanical ventilation, end of surgery to RRT time, AKI to RRT time, presence of postoperative bleeding, presence of hypoxemia, diagnosis of low cardiac output syndrome (LCOS), development of pulmonary infection and (or) sepsis, need for re-exploration, use of an aortic balloon pump (IABP), transfusion requirement for red blood cells (RBC) and (or) plasma. The peak of blood glucose and the peak of lactate on the first postoperative day were selected as the highest values of the test results within 24 h of the first postoperative day. The peak value of lactate and blood glucose before RRT was within 24 h before RRT. If the period from the end of operation to RRT was <24 h, the worst value from the end of the operation to RRT was taken. Three commonly used critical care scores were calculated on admission to the ICU on the first postoperative day and initiation of RRT: the APACHE III score as the base score, the SOFA score as the organ function score, and the VIS as the hemodynamic score. We systematically compared these scores to help clinicians choose a more representative scoring system.

### Definitions

According to the 2012 KIDGO acute kidney injury grading scale, Grade 1 represents an increase in the serum creatinine value 1.5–1.9 times the baseline value and (or) a continuous 6–12 h urine output <0.5 ml/kg; Grade 2 is serum creatinine 2.0–2.9 times the baseline value and (or) urine output <0.5 ml/kg over 12 consecutive h; Grade 3 is serum creatinine more than three times the baseline value and (or) serial 24 h urine output <0.3 ml/kg.

We defined patient short-term survival as 90 days' postoperative survival; in addition, 30 days' survival and mortality at hospital discharge were assessed.

The VIS is the integration of all vasoactive drugs according to the dose (14):


$$\begin{array}{l} \text{VIS}=\text{Dopamine}[\mu \text{g/}(\text{kg}\cdot \text{min})]\text{ + Dobutamine}[\mu \text{g/}(\text{kg}\cdot \text{min})]\\ \;+\;10\times \text{ Milrinone}[\mu \text{g/}(\text{kg}\cdot \text{min})]\text{ + 100}\times \text{ Epinephrine}\\ \;[\mu \text{g/}(\text{kg}\cdot \text{min})]\text{ + 100}\times \text{ Norepinephrine}[\mu \text{g/}(\text{kg}\cdot \text{min})]\\ \;+100\times \text{ Isoproterenol }[\mu \text{ g/}(\text{kg}\cdot \text{min})]\text{ + 10000}\times \\ \text{ Vasopression}[\text{U/}(\text{kg}\cdot \text{min})]. \end{array}$$

More information on the APACHE III and SOFA scores is presented in the discussion section.

The diagnostic criteria for LCOS are: (1) IABP requirement to maintain hemodynamic stability from the end of cardiac surgery to post-ICU hospitalization, and (2) requirement for high-dose vasoactive inotropic agents (dopamine, dobutamine, epinephrine, norepinephrine, etc.) to maintain systolic blood pressure >90 mmHg and cardiac output >2.2 L·min^−1^·m^−2^, after optimizing preload and afterload, as well as correcting all electrolyte and blood gas abnormalities (15).

The bleeding diagnostic criteria are: (1) Intracranial hemorrhage within 48 h of the perioperative period; (2) necessity for an additional operation to control bleeding after the end of the sternotomy; (3) requirement for transfusion of more than 5 U of whole blood or concentrated RBC over 48 h; (4) chest catheter drainage >2 L over 24 h (16).

The diagnosis of lung infection is based on the Guidelines for the Diagnosis and Treatment of Chinese Adult Hospital Acquired Pneumonia and Ventilator-Associated Pneumonia, mainly based on the patient's clinical symptoms, sputum culture, blood culture, physical signs, and blood gas analysis results. Chest X-ray, CT results, etc. are also used as a basis for diagnosis (17).

The diagnostic criteria for sepsis are: Clinical symptoms such as frequent chills and high fever, accompanied by jaundice, anemia, hepatosplenomegaly, petechiae in the skin and mucous membranes, and increased white blood cell and neutrophil counts (18).

To diagnose hypoxemia: SpO_2_ >95% is normal, SpO_2_ 90–95% is hypoxia, SpO_2_ <90% is hypoxemia, and SpO_2_ <80% is moderate to severe hypoxemia (19).

### Statistical Analyses

The data were analyzed using IBM SPSS Statistics for Windows, version 23.0 (IBM Corp., Armonk, NY, USA). Measures of continuous variables that met normal distribution were used as (x ± SD) deviations were indicated, and comparisons between two groups were performed with independent samples *T*-tests. Measurements that did not follow a normal distribution were expressed as medians and quartiles, and comparisons between groups were performed using the Mann–Whitney *U*-test. Data for categorical variables were expressed using the number of cases and percentages, χ^2^ test for comparison. The variables with *P* < 0.10 in the univariable regression analysis and the indicators that had significant significance in previous studies were included in the multivariable logistic regression analysis to determine the independent risk factors for prognosis. The receiver operating characteristics (ROC) curve was constructed to analyze the predictive value of comparing the three scores for the risk of death from RRT in CS-AKI, calculating the area under the receiver operating characteristics curve (AUC_ROC_). The AUC_ROC_ among the three types of scores were compared by calculating the Youden Index and *Z*-test; a difference of *P* < 0.05 was considered statistically significant.

## Results

### Population Characteristics

The 90 patients were divided into a survival group (*n* = 36) and a death group (*n* = 54) according to whether they were alive for 90 days. The mortality rate within 90 days was 60%. Among the 90 patients, 56 were males and 34 were females. The average age of males was (57.23 ± 12.00) years, and the average age of females was (58.83 ± 9.39) years. The results of univariable analysis of preoperative data showed that there was no statistical difference in preoperative-related indexes (*P* > 0.05) (Table 1).

**Table 1:** Univariable analysis of preoperative and intraoperative data.

**Project**	**Survival group**	**Death group**	***t*** **/χ2/z**	***P***
	**(***n*** = 36)**	**(***n*** = 54)**		
Sex /male, ***n*** (%)	24 (66.7)	32 (62.2)	0.504	0.478
Age, years	56.86 ± 11.30	58.48 ± 10.95	0.679	0.499
Body weight, kg	65.44 ± 13.53	62.36 ± 14.13	1.031	0.305
Height, cm	165.72 ± 9.07	163.86 ± 8.48	0.992	0.324
BMI, kg/m^2^	23.69 ± 3.86	23.11 ± 4.43	0.647	0.519
Hypertension, ***n*** (%)	18 (50.0)	25 (46.3)	0.119	0.730
Diabetes mellitus, ***n*** (%)	0	6 (11.1)	–	–
History of cardiac surgery, ***n*** (%)	5 (13.9)	11 (20.4)	0.621	0.431
LVEF, %	57.53 ± 9.02	55.02 ± 8.30	1.351	0.180
HB, g/L	136.56 ± 31.12	131.63 ± 19.65	0.921	0.360
WBC, × 10^9^/L	8.89 ± 4.31	7.69 ± 4.43	1.283	0.203
PLT, × 10^9^/L	179.28 ± 76.40	176.89 ± 65.04	0.159	0.874
Albumin, g/L	39.65 ± 5.06	40.84 ± 5.23	1.068	0.288
TB, μmmol/L	19.45 ± 13.15	21.90 ± 12.81	0.878	0.382
ALT, U/L	33.33 ± 29.79	29.26 ± 19.22	0.789	0.432
AST, U/L	35.58 ± 24.69	31.43 ± 18.16	0.920	0.360
Urea nitrogen, μmmol/L	6.84 ± 2.07	7.78 ± 6.21	0.870	0.387
Creatinine, mmol/L	76.30 ± 19.75	78.31 ± 25.50	0.400	0.690
eGFR, ml/min	97.28 ± 15.94	91.26 ± 18.29	1.602	0.113
Operative time, h	7.49 ± 2.19	7.02 ± 2.49	0.916	0.362
Cardiopulmonary bypass time, min	190.8 (143.0)	160.8 (76.0)	1.582	0.114
Cross clamp time, min	127.8 (102.0)	124.2 (51.0)	0.644	0.520
Transfusion of red blood cells, U	6.35 ± 4.883	5.07 ± 4.24	1.330	0.187
Plasma transfused, ml	759.72 ± 617.93	695.37 ± 635.08	0.476	0.635
Intraoperative urine output, ml	855.71 ± 623.93	936.23 ± 726.88	0.537	0.592

*Values are mean ± SD, median (interquartile range), or percentages*.

*BMI, body mass index; LVEF, left ventricular ejection fraction; HB, hemoglobin; WBC, white blood cell; PLT, platelet; TB, total bilirubin; ALT, alanine transaminase; AST, glutamic oxalacetic transaminase; eGFR, glomerular filtration rate*.

For the intraoperative data, there was no significant difference in the operation time, cardiopulmonary bypass time, cross clamp time, intraoperative RBC infusion, intraoperative plasma infusion, or intraoperative urine output (*P* > 0.05) (Table 1).

The lowest MAP [66.00 (15.50)] on the first postoperative day in the survival group was significantly different compared with the lowest MAP [59.83 (11.80)] on the first postoperative day in the death group (*P* < 0.05). There was a significant difference (*P* < 0.05) in the peak pre-RRT lactate [2.23 (2.45)] in the survival group compared with the peak pre-RRT lactate [5.90 (8.02)] in the death group. There was a significant difference in the APACHE III score [52.00 (21.50)] on the first postoperative day in the survival group compared with the APACHE III score [76.50 (41.80)] on the first postoperative day in the death group (*P* < 0.05).The VIS (15.38 ± 7.18) on the first postoperative day in the survival group was significantly lower than the VIS (22.33 ± 12.61) on the first postoperative day in the death group, and the APACHE III score [53.00 (22.50)] at the start of RRT in the survival group was significantly different compared with the APACHE III score [80.00 (34.30)] at the start of RRT in the death group (*P* < 0.05).The VIS [14.30 ± 8.58] at the start of RRT in the survival group was significantly lower than that [25.93 ± 12.63] at the start of RRT in the death group (Table 2).

**Table 2:** Clinical parameters on the first day after operation and before renal replacement therapy.

**Factors**	**Survival group**	**Death group**	***t*** **/χ2/** ***z***	***p***
	**(***n*** = 36)**	**(***n*** = 54)**		
The first postoperative day (POD1)				
HB, g/L	102.03 ± 18.01	102.84 ± 21.80	0.185	0.854
WBC, × 10^9^/L	13.71 ± 4.98	14.07 ± 5.23	0.322	0.748
PLT, × 10^9^/L	91.33 ± 50.90	97.80 ± 53.60	0.572	0.569
ALB, g/L	32.21 ± 6.50	33.94 ± 7.67	1.115	0.268
TB, μmmol/L	32.86 (30.61)	36.11 (26.01)	0.189	0.850
BUN, μmmol/L	11.86 ± 5.19	10.63 ± 4.65	1.172	0.245
Cr, mmol/L	140.48 ± 84.91	110.48 ± 63.49	1.916	0.059
eGFR, ml/min	60.97 ± 33.74	73.50 ± 32.45	1.766	0.081
CRP, g/L	37.30 (63.92)	28.73 (39.12)	1.557	0.120
MAP, mmHg	66.00 (15.50)	59.83 (11.80)	2.418	0.016^*^
Peak blood glucose, mmol/l	13.37 ± 3.45	12.86 ± 3.46	0.688	0.493
Peak lactate, mmol/l	6.08 ± 3.75	7.32 ± 4.70	1.289	0.201
APACHEIII score	52.0 (21.50)	76.50 (41.80)	4.638	<0.001^*^
VIS score	15.38 ± 7.18	22.33 ± 12.61	2.999	0.004^*^
Before RRT				
HB, g/L	97.92 ± 21.68	101.69 ± 19.81	0.851	0.397
WBC, × 10^9^/L	16.40 ± 5.90	17.80 ± 15.87	0.505	0.615
PLT, × 10^9^/L	89.05 ± 60.93	94.87 ± 67.83	0.415	0.679
ALB, g/L	37.04 ± 6.23	38.28 ± 7.67	0.811	0.420
TB, μmmol/L	47.92 ± 33.54	45.98 ± 33.78	0.268	0.789
BUN, μmmol/L	25.94 ± 15.56	30.08 ± 21.85	0.984	0.328
Cr, mmol/L	296.03 ± 149.17	266.49 ± 146.80	0.929	0.355
eGFR, ml/min	28.22 ± 22.96	29.76 ± 22.39	0.316	0.753
CRP, g/L	92.97 (106.76)	75.19 (154.19)	1.036	0.300
MAP, mmHg	75.98 ± 12.39	70.34 ± 16.12	1.777	0.079
Peak blood glucose, mmol/l	11.11 ± 2.97	7.82 ± 12.35	1.100	0.275
Peak lactate, mmol/l	2.23 (2.45)	5.90 (8.02)	3.675	<0.001^*^
Oliguria time before RRT, h	6.57 ± 11.22	7.82 ± 12.35	0.484	0.630
APACHEIII score	53.00 (22.50)	80.00 (34.30)	5.866	<0.001^*^
VIS score	14.30 ± 8.58	25.93 ± 12.63	4.239	<0.001^*^

*Values are mean ± SD, median (interquartile range), or percentages*.

* *The difference is statistically significant; HB, hemoglobin; PLT, platelet; ALB, albumin; TB, total bilirubin; BUN, blood urea nitrogen; Cr, creatinine; eGFR, glomerular filtration rate; CRP, C-reactive protein; MAP, mean arterial pressure; APACHEIII, acute physiology and health score; SOFA, sequential organ failure assessment; VIS, vasoactive-inotropic score; RRT, renal replacement therapy*.

The mortality rate at discharge was 54.4%, and the mortality rate within 30 days was 51.1%. In this study, 43 patients (47.8%) had preoperative hypertension, 6 patients (6.7%) had preoperative diabetes, and 16 patients (17.8%) had undergone cardiac surgery. Of the 90 patients, 57 patients (63.3%) underwent valve replacement surgery, 9 patients (10%) underwent valve replacement combined with aorta–coronary artery bypass surgery, 4 patients (4.4%) underwent aorta–coronary artery bypass surgery, and 19 patients (21.1%) underwent aortic and great vessel surgery. In the postoperative data, postoperative bleeding (27 patients) and postoperative LCOS (39 patients) in the death group were significantly higher than those in the survival group. Among the 90 patients, 46 patients (51.1%) were treated with CRRT, and 44 patients (48.9%) were treated with IRRT. Nineteen patients (21.1%) were treated with heparin, 24 patients (26.7%) were treated with sodium citrate, and 47 patients (52.2%) received no anticoagulation (Table 3).

**Table 3:** Postoperative relevant clinical indicators with dialysis-dependent acute kidney injury after cardiac surgery.

**Factors**	**Survival group**	**Death group**	***t*** **/χ2/** ***z***	***P***
	**(***n*** = 36)**	**(***n*** = 54)**		
Hypoxemia, *n* (%)	15 (41.7)	24 (44.4)	0.068	0.794
Pulmonary infection, *n* (%)	13 (36.1)	29 (53.7)	2.686	0.101
Hemorrhage, *n* (%)	8 (22.2)	27 (50)	7.013	0.008^*^
LCOS, *n* (%)	18 (50.0)	39 (72.2)	4.593	0.032^*^
Septicemia, *n* (%)	1 (2.8)	6 (11.1)	2.091	0.148
Re-exploration, *n* (%)	2 (5.6)	7 (13.0)	1.317	0.251
IABP, *n* (%)	4 (11.1)	10 (18.5)	0.902	0.342
Duration of mechanical ventilation, days	6.33 ± 10.16	5.64 ± 6.21	0.398	0.692
Duration of RRT support, days	8.91 ± 7.75	8.57 ± 8.62	0.189	0.850
End of surgery to time of RRT, days	3.83 ± 4.74	5.51 ± 6.65	1.306	0.195
AKI to RRT time, h	65.81 ± 91.94	99.78 ± 151.62	1.204	0.232
Postoperative RBC transfusion, U	11.36 ± 7.25	14.13 ± 11.81	1.256	0.212
Postoperative infusion of plasma, ml	2063.9 ± 2010.2	2840.3 ± 2994.6	1.363	0.176
Surgical approach				
Valve replacement, *n* (%)	19 (52.8)	38 (70.4)		0.064
Valve replacement + bypass, *n* (%)	2 (5.6)	7 (13.0)		
Bypass, *n* (%)	2 (5.6)	2 (3.7)		
Aortic surgery, *n* (%)	12 (33.3)	7 (13.0)		
Other procedures, *n* (%)	1 (2.8)	0 (0)		
AKI grading				
Grade 1, *n* (%)	4 (11.4)	10 (18.5)		0.448
Grade 2, *n* (%)	10 (27.8)	10 (18.5)		
Grade 3, *n* (%)	22 (61.1)	34 (63.0)		
Anticoagulation modality				
No anticoagulation, *n* (%)	19 (52.8)	28 (51.9)		0.636
Heparin, *n* (%)	6 (16.7)	13 (24.1)		
Sodium citrate, *n* (%)	11 (30.6)	13 (24.1)		
Pattern				
CRRT, *n* (%)	18 (50.0)	28 (51.9)		0.863
IRRT, *n* (%)	18 (50.0)	26 (48.1)		
Death at 30 days, *n* (%)	0 (0)	46 (85.2)		
Death at hospital discharge, *n* (%)	0 (0)	49 (90.7)		
MODS at discharge	2 (5.6)	54 (96.4)		P < 0.001
Cr at discharge, mmol/L	177.83 ± 142.86	188.78 ± 142.46	0.356	0.722
eGFR, ml/min	59.56 ± 38.12	59.31 ± 45.68	0.026	0.979

*Values are mean ± SD, median (interquartile range), or percentages*.

* *The difference is statistically significant. LCOS, low cardiac output syndrome; IABP, intra-aortic balloon pump; CRRT, continuous renal replacement therapy; IRTT, intermittent renal replacement therapy; MODS, multiple organ dysfunction syndrome; Cr, creatinine; eGFR, glomerular filtration rate*.

### ROC Curve Group Comparison Results

**Figure 1:**
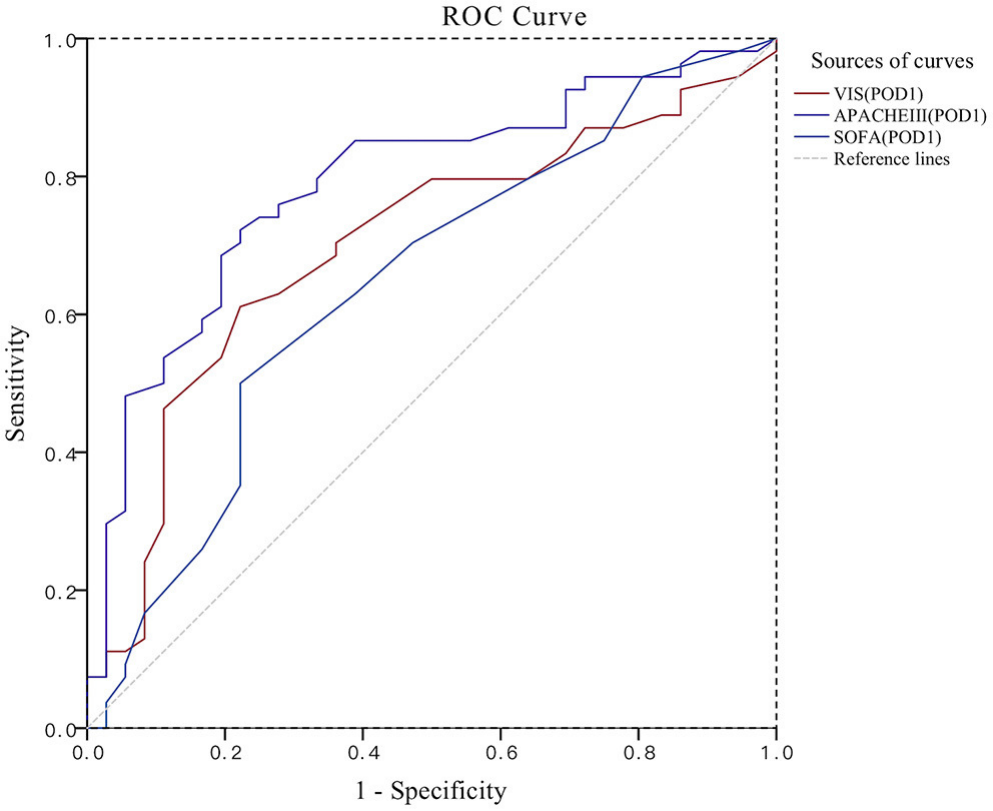
ROC plot for the comparison of three scores. POD1, the first postoperative day; APACHEIII, acute physiology and health score; SOFA, sequential organ failure assessment; VIS, vasoactive-inotropic score.

**Figure 2:**
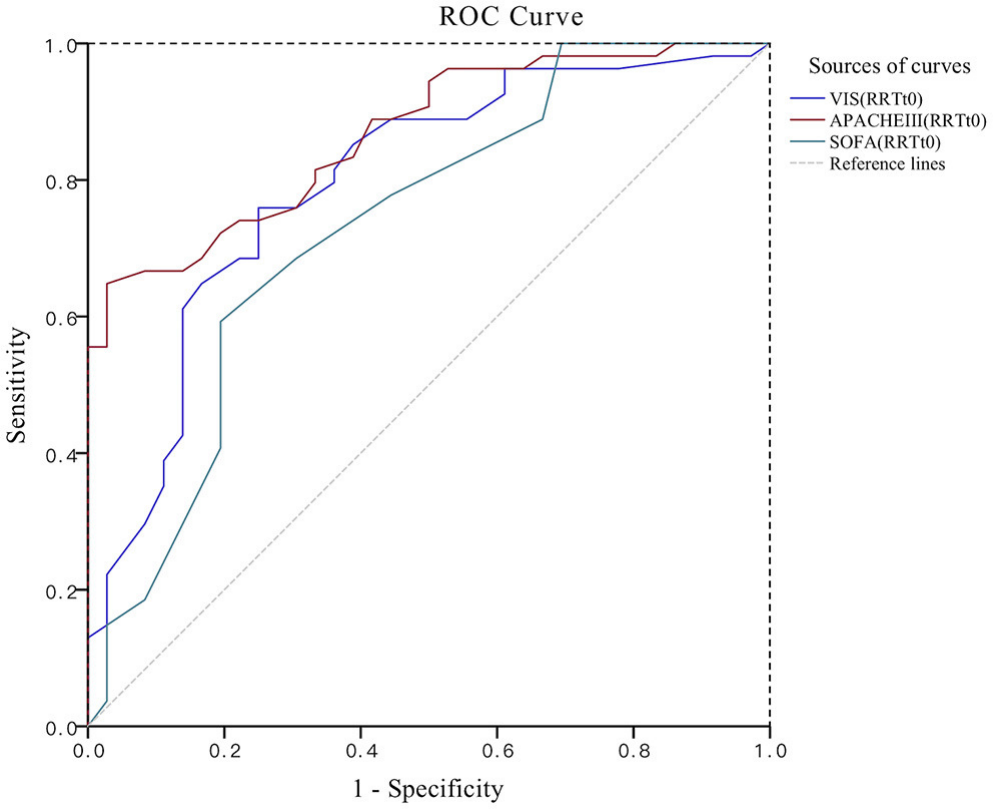
ROC plot for the comparison of three scores. RRT, renal replacement therapy; RRTt_0_, the start of RRT; APACHEIII, acute physiology and health score; SOFA, sequential organ failure assessment; VIS, vasoactive-inotropic score.

The APACHEIII score, VIS, and SOFA score at the beginning of RRT were established on the first day after surgery to establish the ROC curve to evaluate the prediction of the risk of death from acute AKI after cardiac surgery (Figures 1, 2). The APACHEIII score AUC_ROC_ on the first day after surgery was 0.790, the 95% CI (0.694, 0.885), and the AUC-value of the VIS on the first postoperative day was 0.700, the 95% CI (0.589, 0.811). On the first day after surgery, the SOFA score was the lowest (0.060), the 95% CI (0.527, 0.763). At the beginning of RRT, the APACHEIII score AUC_ROC_ was 0.866, the 95% CI (0.795, 0.937). At the beginning of RRT, the VIS AUC_ROC_ was 0.796, the 95% CI (0.700, 0.892). The SOFA score AUC_ROC_ at the beginning of RRT was 0.732, the 95% CI (0.623, 0.842) (Table 4). The cut-off value was calculated by calculating the maximum cut-off point of the Youden Index as a diagnostic cut-off value for predicting patient death. The AUC-value of the APACHE III score (0.866) was highest at the start of RRT, with the highest Youden Index of 0.620, the highest specificity of 97.2% and a cut-off value of 75. The VIS at the start of RRT had the lowest specificity of 0.750 and the highest sensitivity of 0.759, with a Youden Index of 0.509 and a cut-off value of 18.9 (Table 4). On the first postoperative day, the SOFA-value had the lowest sensitivity, which was 0.500, the specificity was 0.778, and the cut-off value was 11.5. The AUC-value of the six scoring indicators was tested with the *Z*-test, and the APACHE III score at the beginning of RRT was significantly different from the SOFA score at the beginning of RRT (Z = 2.013, *P* = 0.044) (*P* < 0.05). The APACHE III score at the beginning of RRT was significantly different from the VIS on the first day after surgery (Z = 2.462, *P* = 0.014) (*P* < 0.05). The APACHEIII score before RRT was compared with the SOFA score on the first day after surgery (*Z* = 3.158, *P* = 0.002), and the difference was statistically significant (*P* < 0.05); the difference between the other two scores was not statistically significant (Table 5).

**Table 4:** ROC curve measures.

**Score**	**AUC**_***ROC***_	**SE**	**P^b^**	**95%CI**	**Cut-off**	**Sensitivity**	**Specificity**	**Youden**
**POD1**								
APACHEIII	0.790	0.049	<0.001	0.694–0.885	62.0	0.722	0.778	0.500
VIS	0.700	0.057	0.001	0.589–0.811	19.5	0.611	0.778	0.389
SOFA	0.645	0.060	0.020	0.527–0.763	11.5	0.500	0.778	0.278
**RRTt**_**0**_								
APACHEIII	0.866	0.036	<0.001	0.795–0.937	75	0.648	0.972	0.620
VIS	0.796	0.049	<0.001	0.700–0.892	18.9	0.759	0.750	0.509
SOFA	0.732	0.056	<0.001	0.623–0.842	13.5	0.593	0.806	0.399

b *Null hypothesis: true Region = 0.5*.

*POD1, The first postoperative day; APACHEIII, acute physiology and health score; VIS, vasoactive-inotropic score; RRT, renal replacement therapy; RRTt_0_, the initiate of RRT; SOFA, sequential organ failure assessment*.

**Table 5:** Results of pairwise score comparison.

**Score**	**Compare**	***SE***	***Z***	***P***
APACHEIII(POD1)				
	vs. VIS(POD1)	0.006	1.197	0.231
	vs. APACHEIII(RRTt_0_)	0.004	1.250	0.211
	vs. VIS(RRTt_0_)	0.005	0.087	0.931
	vs. SOFA(RRTt_0_)	0.006	0.779	0.438
	vs. SOFA(POD1)	0.006	1.872	0.077
VIS(POD1)				
	vs. APACHEIII(RRTt_0_)	0.005	2.462	0.014^*^
	vs. VIS(RRTt_0_)	0.006	1.277	0.202
	vs. SOFA(RRTt_0_)	0.006	0.400	0.689
	vs. SOFA(POD1)	0.007	0.665	0.506
APACHEIII(RRTt_0_)				
	vs. VIS(RRTt_0_)	0.004	1.151	0.250
	vs. SOFA(RRTt_0_)	0.004	2.013	0.044^*^
	vs. SOFA(POD1)	0.005	3.158	0.002
VIS(RRTt_0_)				
	vs. SOFA(RRTt_0_)	0.006	0.860	0.390
	vs. SOFA(POD1)	0.006	1.949	0.051
SOFA(RRTt_0_)				
	vs. SOFA(POD1)	0.007	1.060	0.289

* *The difference is statistically significant. APACHEIII, acute physiology and health score; POD1, the first postoperative day; VIS, vasoactive-inotropic score; RRT, renal replacement therapy; RRTt_0_, the initiate of RRT; SOFA, sequential organ failure assessment*.

### Multivariate Logistic Regression Analysis Results

Variables with *P* < 0.1 in the univariable regression analysis (lowest MAP on the first postoperative day, MAP before RRT, peak lactate before RRT, postoperative bleeding, postoperative LCOS, creatinine value on the first postoperative day, glomerular filtration rate, APACHE III score on the first postoperative day, VIS on the first postoperative day, APACHE III score at the start of RRT, VIS at the start of RRT) as well as indicators that were significant in previous studies (time from AKI to RRT) were included in the multivariable binary logistic regression equation. Variant assignments are presented in Table 6. We found that a high APACHE III score at the start of RRT (OR: 1.228, 95% CI: 1.079–1.397), high VIS at the start of RRT (OR: 1.147, 95% CI: 1.021–1.290), low MAP before RRT (OR: 1.170, 95% CI: 1.050–1.303), high lactate value before RRT (OR: 1.552, 95% CI: 1.032–2.333), and long time from AKI to initiation of RRT (OR: 1.014, 95% CI: 1.002–1.027) were independent risk factors for mortality in patients with acute kidney injury undergoing RRT after cardiac surgery (Table 7).

**Table 6:** Results of variable assignment.

**Factors**	**Variables**	**Assignments**
90 days survival after surgery	Y	NO = 1, YES = 0
MAP before RRT	X_1_	Quantitative data
APACHEIII(RRTt_0_)	X_2_	Quantitative data
VIS(RRTt_0_)	X_3_	Quantitative data
Peak lactate before RRT	X_4_	Quantitative data
AKI to RRT time	X_5_	Quantitative data
LCOS	X_6_	YES = 1, NO = 0
Hemorrhage	X_7_	YES = 1, NO = 0
Cr (POD1)	X_8_	Quantitative data
eGFR (POD1)	X_9_	Quantitative data

*MAP, mean arterial pressure; RRT, renal replacement therapy; APACHEIII, acute physiology and health score; RRTt_0_: day before RRT; VIS, vasoactive-inotropic score; AKI, acute kidney injury; LCOS, low cardiac output syndrome; Cr, creatinine; POD1, the first postoperative day; eGFR, glomerular filtration rate*.

**Table 7:** Results of multivariable logistic regression analysis.

**Factors**	***B***	***SE***	**Wals**	**Exp** **(***B***)(95%CI)**	***P***
MAP before RRT	0.157	0.055	8.103	1.170 (1.050–1.303)	0.004^*^
APACHEIII(RRTt_0_)	0.205	0.066	9.744	1.228 (1.079–1.397)	0.002^*^
VIS(RRTt_0_)	0.138	0.060	5.303	1.147 (1.021–1.290)	0.021^*^
Peak lactate before RRT	0.439	0.208	4.462	1.552 (1.032–2.333)	0.035^*^
AKI to RRT time	0.014	0.006	4.808	1.014 (1.002–1.027)	0.028^*^
LCOS	0.620	0.970	0.408	1.858 (0.278–12.438)	0.523
Hemorrhage	0.283	0.993	0.081	1.327 (0.189–9.299)	0.776
Cr (POD1)	−0.002	0.009	0.042	0.998 (0.980–1.016)	0.837
eGFR (POD1)	0.025	0.025	0.972	1.025 (0.976–1.076)	0.324
APACHEIII(POD1)	0.003	0.024	0.012	1.003 (0.956–1.051)	0.912
VIS(POD1)	0.036	0.049	0.541	1.037 (0.941–1.142)	0.462

*Logistic regression analysis was used to determine the risk factors of death*.

* *The difference is statistically significant; MAP, mean arterial pressure; RRT, renal replacement therapy; APACHEIII, acute physiology and health score; RRTt_0_, day before RRT; VIS, vasoactive-inotropic score; AKI, acute kidney injury; LCOS, low cardiac output syndrome; Cr, creatinine; POD1, the first postoperative day; eGFR, glomerular filtration rate*.

## Discussion

Currently, many methods are applied clinically to reduce the occurrence of postoperative AKI, but the mortality of postoperative AKI remains high (20, 21). The severity of postoperative AKI affecting the heart directly influences patient prognosis. The current proportion of patients with postoperative renal injury requiring continuous RRT is small (1). The present study found a 90-day survival rate of 40% after RRT in patients with new-onset AKI after surgery. According to the literature, the mortality rate of patients with AKI requiring RRT exceeds 50% (22). This is consistent with the 90-day mortality rate (60%) in this study, in which five (12.2%) of the patients who survived to hospital discharge had a poor 90-day postoperative prognosis.

At present, little is known about the prognostic factors affecting the development of CS-AKI treated with RRT. Among the numerous observations included in this study, multivariable logistic regression revealed that low MAP before RRT was an independent risk factor for the outcome of RRT in patients with CS-AKI. MAP on the first postoperative day was significantly lower in the death group than in the survival group in univariable analysis, indicating that postoperative hypotension and hypotension before RRT significantly increase the risk of short-term mortality in patients with postoperative renal failure treated with RRT. Postoperative hypotension significantly increases length of stay in the ICU and prolongs tracheal intubation in patients who die after cardiac surgery (23). A retrospective study has shown that preoperative renal insufficiency and low postoperative blood pressure are both the most important risk factors for the development of AKI after cardiac surgery (24). Cardiac surgery is highly traumatic, and the postoperative period is prone to excessive bleeding, LCOS, and hypovolemia. These conditions are most directly manifested in low blood pressure values; blood perfusion of the kidney will be significantly reduced, preferentially supplying important organs such as heart and brain, causing ischemic perfusion injury, and leading to multiple organ failure.

Although there is still no clear guideline for RRT in CS-AKI, there is a consensus that early RRT can improve the prognosis of patients (25–29). In this study, although the univariable analysis of the interval between AKI and RRT showed that there was no statistical significance between the survival group and the death group, the occurrence of AKI in the death group was associated with later initiation of RRT. Combined with previous studies and professional knowledge, we still include the AKI to RRT interval in the multivariable analysis. Interestingly, the final result suggests that the AKI to RRT interval is an independent risk factor that affects the short-term death of patients. This indicates that after AKI occurs following cardiac surgery, if timely intervention is not available, the condition will further deteriorate, and it will be difficult to achieve satisfactory treatment results in the later stage. The timely and effective development of RRT will significantly reduce the mortality of CS-AKI patients.

The high lactate level before RRT is another independent risk factor found in this study to affect the mortality of patients with CS-AKI. In many studies, high lactate levels directly affect the mortality risk of patients with severe AKI (25, 30). Serum lactic acid reflects the severity of organ failure, which has been confirmed in a number of studies (31). This study also confirmed that the high lactate value before RRT will directly affect the short-term survival of patients. The high or low lactate value can help us choose the timing of RRT and improve the prognosis of patients.

The other main purpose of this study was to analyze and compare three different types of scores: APACHE III score, SOFA score, and VIS. Several updated versions of the critical care score have appeared successively in the 1990s and are increasingly being validated in the clinic (32–34). These include the updated APACHE III generation scoring system that predicts patients' conditions more comprehensively, meticulously, reasonably, and accurately (35). Knaus et al. (34) in 1991 proposed the APACHE III scoring scheme on the basis of a two-generation scoring scheme with a total of 17 items included in the physiology score. New variables were added to the APACHE II score, such as serum creatinine, urine output reflecting renal function, serum albumin, total bilirubin reflecting hepatic function, and blood glucose. These additions enabled a more accurate extrapolation of the magnitude of risk for death. A German study, studying 531 patients in their ICUs, found that the mortality predicted by the APACHE III score and the actual patients' mortality differed by only 0.2% (36). In the current study, the highest specificity of the APACHE III score was found at the start of RRT, with a significantly higher AUC (0.866) than the SOFA score (0.796) and the VIS (0.732), and the AUC *Z*-test showed that the APACHE II score on the day before RRT was significantly better than the SOFA score. Moreover, the APACHE III score at the start of RRT (OR: 1.228, 95% CI: 1.079–1.397) was an independent risk factor for poor outcome in patients undergoing RRT after CS-AKI. The APACHE III score is more accurate than the SOFA score and VIS and may predict the course of disease development in patients with CS-AKI. Furthermore, a unique advantage of the APACHE III score is reflected in its most well-established inclusion of indicators of renal function, allowing timely monitoring of changes. The APACHE III score is mainly based on language, pain response, and motor function when assessing central nervous function. Patients after cardiac surgery are largely sedated in the early stages of ICU admission, and the use of the Glasgow Coma Scale in APACHE II biases the results and does not precisely reflect the physiological status. The APACHE III score emphasizes the use of the original physiological indexes, and the evaluation of vital signs is more precise. According to this and previous studies, postoperative hypotension is a significant risk factor for RRT in patients with acute renal failure after cardiac surgery (24, 37). Of the three scores (APACHE III, SOFA, and VIS), only the APACHE III score utilizes MAP, which may also be the reason why APACHE III is a better predictor of mortality than the SOFA score and VIS.

The sequential organ failure score (SOFA score) is currently widely used in the evaluation of organ function in critically ill patients, and previous studies have shown that the SOFA score has a high value for predicting patient mortality after cardiac surgery (38). Skarupskiene (37), in a single-center retrospective study showed that the SOFA score is an accurate method to predict mortality outcomes in patients with AKI requiring RRT after cardiac surgery. A cardiac care unit patient outcome score study by Argyriou (39), found that the APACHE II evaluation performed better than the SOFA score. In this study, the AUC_ROC_-value of the SOFA score at the start of RRT was 0.732, and the Youden Index was 0.399, which could reflect the prognosis of patients undergoing RRT, but was less predictive than the APACHE III score and VIS. The AUC_ROC_-value of the SOFA score on the first postoperative day was 0.645, which was <0.700, indicating that the SOFA score on the first postoperative day could not accurately predict the probability of death in patients undergoing RRT. The possible reason was that multiple organ damage was at an early stage on the first postoperative day, and the SOFA score at this time could not reflect the injury severity. After a *Z*-test of the three scoring AUC-values, we found that the SOFA score was lower at the start of RRT compared with the APACHE III score at the start of RRT (*Z* = 2.013, *P* = 0.044), but there was no statistical difference between the VIS at the start of RRT (*Z* = 0.860, *P* = 0.390). The SOFA score at the start of RRT reflected, to some extent, the risk of mortality in patients with CS-AKI on RRT, but the diagnostic value was moderate.

The VIS is a vasoactive drug score, first proposed by Gaies et al. (14). The content of the score is calculated by calculating the dose of dopamine, dobutamine, milrinone, norepinephrine, and epinephrine in the patient's body. It is of unique value in cardiac surgery, when patients in the early postoperative period are prone to develop LCOS; dopamine, norepinephrine, and others can promote blood redistribution. This is accomplished by constriction of peripheral blood vessels and maintenance of blood supply to important organs, but it may easily cause insufficient perfusion of peripheral organs. Yamazaki et al. (40) showed that the VIS was able to accurately predict postoperative cardiac mortality. A study by Koponen et al. (41) showed that the VIS had a higher predictive accuracy for postoperative cardiac death than the APACHE II score, but it was not significantly different from the SOFA score. In this study, the number of patients with postoperative LCOS in the survival group was significantly lower than that in the death group, and the occurrence of postoperative low cardiac output would increase the dosage of vasoactive drugs. Low MAP before RRT is an important prognostic factor in patients undergoing RRT, and similarly increases the use of vasopressors such as dopamine and epinephrine, with a concomitant increase in the VIS. In addition, the VIS at the start of RRT (OR: 1.147, 95% CI: 1.021–1.290) was another independent risk factor affecting the performance of RRT in patients with CS-AKI. The AUC-value of the VIS at the start of RRT was 0.796, which was second only to the APACHE III score (AUC_ROC_ = 0.866). The above results indicate that the ability of VIS to predict the postoperative course of patients at the beginning of RRT is weaker than the APACHE III score, although it still has a better value for predicting the risk of death.

In recent years, some scholars have suggested that the treatment indication for CRRT should be changed from maintaining and improving renal function to functional support to systemic organs (42, 43). Evaluation of the overall function of postoperative patients can help us to initiate RRT as early as possible and effectively improve patient outcomes. A comparison revealed that the APACHE III score on the first postoperative day (AUC_ROC_ = 0.790) was significantly higher than the VIS (AUC_ROC_ = 0.700), indicating that the APACHE III score on the first postoperative day as an early indicator can reflect the patient's treatment prognosis to a certain extent and help us to determine the timing of early RRT.

## Limitations

There are several limitations in our study. This study is a single-center retrospective study, with fewer samples and a certain degree of bias, which increases the risk of “type I errors.” We look forward to a multicenter study with a larger number of patients to propose a more accurate scoring system. The shortfalls of this study also include AKI requiring RRT after cardiac surgery—both the cause and the effect of a very complex pathophysiologic process, and the risk of having confounders in the multivariable analysis is quite high.

## Conclusions

In conclusion, the APACHE III score and VIS had high value for predicting the risk of mortality in patients with CS-AKI undergoing RRT; the APACHE III score at the start of RRT had the strongest predictive power, and the APACHE III score on the first postoperative day could be used as an indicator for starting early RRT. Moreover, the APACHE III score, VIS at the start of RRT, MAP before RRT, lactate value before RRT, and time from AKI to the initiation of RRT were the predictors found to influence the performance of RRT in patients with CS-AKI.

## Data Availability Statement

The data analyzed in this study is subject to the following licenses/restrictions: Medical records were obtained from the medical record system of the First Affiliated Hospital of Anhui Medical University, users are not allowed to distribute the raw data, therefore the raw data could not be made publicly available by authors. Requests to access these datasets should be directed to Huiyong Han, 1511366770@qq.com.

## Ethics Statement

The studies involving human participants were reviewed and approved by Fast-Ethical Approval of the First Affiliated Hospital of Anhui Medical University-P2021-13-20. Written informed consent for participation was not required for this study in accordance with the national legislation and the institutional requirements.

## Author Contributions

HH and JD conceived and designed the experiments. HH collected and analyzed the data and wrote the manuscript. SG, ZW, JW, PZ, and QG reviewed and edited the manuscript. All authors contributed to the article and approved the submitted version.

## Funding

This work was supported by a grant from the Cultivation plan of National Natural Science Foundation youth fund of the First Affiliated Hospital of Anhui Medical University (2021Kj06).

## Conflict of Interest

The authors declare that the research was conducted in the absence of any commercial or financial relationships that could be construed as a potential conflict of interest.

## Publisher's Note

All claims expressed in this article are solely those of the authors and do not necessarily represent those of their affiliated organizations, or those of the publisher, the editors and the reviewers. Any product that may be evaluated in this article, or claim that may be made by its manufacturer, is not guaranteed or endorsed by the publisher.
