# Supplementary material for: Prediction of Short-Term Mortality With Renal Replacement Therapy in Patients With Cardiac Surgery-Associated Acute Kidney Injury
Source: Front Cardiovasc Med. 2021 Oct 21;8:738947. doi: 10.3389/fcvm.2021.738947 (PMC8566707; doi:10.3389/fcvm.2021.738947)
Supplement: Supplementary file 1 [file Table_1.DOCX]

APACHEIII

**Pluse, beats/min**

| $\leq$39 | 40-49 | 50-99 | 100-109 | 110-119 | 120-139 | 140-154 | $\geq$155 |
| --- | --- | --- | --- | --- | --- | --- | --- |
| 8 | 5 | 0 | 1 | 5 | 7 | 13 | 17 |

**MAP, mmHg**

| $\leq$39 | 40-59 | 60-69 | 70-79 | 80-99 | 100-119 | 120-129 | 130-139 | $\geq$140 |
| --- | --- | --- | --- | --- | --- | --- | --- | --- |
| 23 | 16 | 7 | 6 | 0 | 4 | 7 | 9 | 10 |

**Temperature,** $\boldsymbol{℃}$

| $\leq$32.9 | 33-33.4 | 33.5-33.9 | 34-34.9 | 35-35.9 | 36-39.9 | $\geq$40 |
| --- | --- | --- | --- | --- | --- | --- |
| 20 | 16 | 13 | 8 | 2 | 0 | 4 |

**Respiratory Rate, beats/min**

| $\leq$5 | 6-11 | 12-13 | 14-24 | 25-34 | 35-39 | 40-49 |
| --- | --- | --- | --- | --- | --- | --- |
| 17 | 8 | 7 | 0 | 6 | 9 | 18 |

* **For patients who are ventilated no points are given for respiratory rates of 6-12.**

**PaO2, mmHg**

| $\leq$49 | 50-69 | 70-79 | $\geq$80 |
| --- | --- | --- | --- |
| 15 | 5 | 2 | 0 |

**A-aDO2*, mmHg**

| <100 | 100-249 | 250-349 | 350-499 | $\geq$500 |
| --- | --- | --- | --- | --- |
| 0 | 7 | 9 | 11 | 14 |

*** Only use A-aDO2 for intubated patients with FiO2**$\boldsymbol{\geq}$**0.5**

**Hematocrit, %**

| $\leq$40.9 | 41-49 | $\geq$50 |
| --- | --- | --- |
| 3 | 0 | 3 |

**WBC,** $\boldsymbol{\times}$**10^9^/L**

| <1.0 | 1.0-2.9 | 3.0-19.9 | 20-24.9 | $\geq$25.0 |
| --- | --- | --- | --- | --- |
| 19 | 5 | 0 | 1 | 5 |

**Creatinine without ARF***

| $\leq$43 | 44-132 | 133-171 | $\geq$172 |
| --- | --- | --- | --- |
| 3 | 0 | 4 | 7 |

*Acute Renal Failure (ARF) is defined as creatinine $\geq$1.5mg/dl and urine output <410 ml/d and no chronic dialysis

**BUN, mmol/L**

| $\leq$6.1 | 6.2-7.1 | 7.2-14.3 | 14.4-28.5 | $\geq$28.6 |
| --- | --- | --- | --- | --- |
| 0 | 2 | 7 | 11 | 12 |

**Urine Output, ml/d**

| $\leq$399 | 400-599 | 600-899 | 900-1499 | 1500-1999 | 2000-3999 | $\geq$4000 |
| --- | --- | --- | --- | --- | --- | --- |
| 15 | 8 | 7 | 5 | 4 | 0 | 1 |

**Na^+^, mmol/L**

| $\leq$119 | 120-134 | 135-154 | $\geq$155 |
| --- | --- | --- | --- |
| 3 | 2 | 0 | 4 |

**Albumin, g/L**

| $\leq$19 | 20-24 | 25-44 | $\geq$45 |
| --- | --- | --- | --- |
| 11 | 6 | 0 | 4 |

**Bilirubin,** $\boldsymbol{\mu}$**mol/L**

| $\leq$34 | 35-51 | 52-85 | 86-135 | $\geq$136 |
| --- | --- | --- | --- | --- |
| 0 | 5 | 6 | 8 | 16 |

**Glucose, mmol/dL**

| $\leq$2.1 | 2.2-3.3 | 3.4-11.1 | 11.2-19.3 | $\geq$19.4 |
| --- | --- | --- | --- | --- |
| 8 | 9 | 0 | 3 | 5 |

**Neurological Abnormalities**

**If patient’ s eyes open spontaneously or to painful/verbal stimulation, use scale:**

| verbal | Oriented, | Confused conversation | Words,  in comp.  sounds | No response |
| --- | --- | --- | --- | --- |
| motor | converses |  |  |  |
| Obeys  verbal commands | 0 | 3 | 10 | 15 |
| Localizes pain | 3 | 8 | 13 | 15 |
| Flexion withdrawal/decorticated rigidity | 3 | 13 | 24 | 24 |
| Decerebrate rigidity/no response | 3 | 13 | 29 | 29 |

**If patient’ s eyes do not open spontaneously or to painful/verbal stimulation, use scale:**

| verbal | Oriented, | Confused conversation | Words,  in comp.  sounds | No response |
| --- | --- | --- | --- | --- |
| motor | converses |  |  |  |
| Obeys  verbal commands |  |  |  | 16 |
| Localizes pain |  |  |  | 16 |
| Flexion withdrawal/decorticated rigidity |  |  | 24 | 33 |
| Decerebrate rigidity/no response |  |  | 29 | 48 |

**Acid-Base Abnormalities**

| PCO2 | <25 | 25~<30 | 30~<35 | 35~<40 | 40~<45 | 45~<50 | 50~<55 | 55~<60 | $\geq$60 |
| --- | --- | --- | --- | --- | --- | --- | --- | --- | --- |
| PH |  |  |  |  |  |  |  |  |  |
| <7.15 | 12 | | | | | | 4 | | |
| 7.15~<7.20 |  |  |  |  |  |  |  |  |  |
| 7.20~<7.25 | 9 | | 6 | | 3 | | 2 | | |
| 7.25~<7.30 |  |  |  |  |  |  |  |  |  |
| 7.30~<7.35 |  |  | 0 | | | 1 | | | |
| 7.35~<7.40 | 5 | |  |  |  | 1 | | | |
| 7.40~<7.45 |  |  |  |  |  |  |  |  |  |
| 7.45~<7.50 |  |  | 0 | 2 | |  |  |  |  |
| 7.50~<7.55 | 3 | | | | 12 | | | | |
| 7.55~<7.60 |  |  |  |  |  |  |  |  |  |
| 7.60~<7.65 | 0 |  |  |  |  |  |  |  |  |
| $\geq$7.65 |  |  |  |  |  |  |  |  |  |

**Age of patient**

| $\leq$44 | 45-59 | 60-64 | 65-69 | 70-74 | 75-84 | $\geq$85 |
| --- | --- | --- | --- | --- | --- | --- |
| 0 | 5 | 11 | 13 | 16 | 17 | 24 |

**Chronic Health Items present on admission**

| AIDS | Hepatic Failure | lymphoma | Metastatic cancer | Leukemia/multiple myeloma | Immunosuppression | Cirrhosis |
| --- | --- | --- | --- | --- | --- | --- |
| 23 | 16 | 13 | 11 | 10 | 10 | 4 |
