# Supplementary material for: Prediction of Short-Term Mortality With Renal Replacement Therapy in Patients With Cardiac Surgery-Associated Acute Kidney Injury
Source: Front Cardiovasc Med. 2021 Oct 21;8:738947. doi: 10.3389/fcvm.2021.738947 (PMC8566707; doi:10.3389/fcvm.2021.738947)
Supplement: Supplementary file 2 [file Table_2.DOCX]

**SOFA**

| system |  | 0 | 1 | 2 | 3 | 4 |
| --- | --- | --- | --- | --- | --- | --- |
| Respiration | PaO2/FiO2, mm Hg(kPa) | $\geq$400(53.5) | <400(53.3) | <300(40) | <200(26.7) with respiratory support | <100(13.3) with respiratory support |
| Coagulation | Platelets, $\times$10^3^/$\mu$L | $\geq$150 | <150 | <100 | <50 | <20 |
| Liver | Bilirubin, $\mu$mol/l | <20.5 | $\leq$34.1 | $\leq$102.5 | $\leq$205.1 | >205.1 |
| Cardiovascular | MAP, mmHg | $\geq$70 | <70 | Dopamine<5 or dobutamine (any dose) | Dopamine<5.1-15 or epinephrine$\leq$0.1 or norepinephrine$\leq$0.1 | Dopamine>15 or epinephrine>0.1 or norepinephrine>0.1 |
| Central nervous system | Glasgow Coma Scale score | 15 | 13-14 | 10-12 | 6-9 | <6 |
| Renal | Creatinine, mg/dl($\mu$mol/l) | <1.2(110) | 1.2-1.9(110-170) | 2.0-3.4(171-299) | 3.5-4.9(300-440) | >5.0(440) |
|  | Urine output, mL/d |  |  |  | <500 | <200 |

Abbreviations: FiO2, fraction of inspired oxygen; MAP, mean arterial pressure; PaO2, partial pressure of oxygen.
